# Evaluation of the effect of 2-(2,4-dihydroxyphenyl)-4*H*-benzofuro[3,2-*d*][1,3]thiazin-4-one on colon cells and its anticancer potential

**DOI:** 10.1007/s00044-018-2223-8

**Published:** 2018-07-25

**Authors:** Arkadiusz Czerwonka, Marta K. Lemieszek, Monika Karpińska, Joanna Matysiak, Andrzej Niewiadomy, Wojciech Rzeski

**Affiliations:** 1 Department of Virology and Immunology, Maria Curie-Skłodowska University, Akademicka 19, Lublin, 20-400 Poland; 2 Department of Medical Biology, Institute of Rural Health, Jaczewskiego 2, Lublin, 20-090 Poland; 3 Institute of Industrial Organic Chemistry, Annopol 6, Warsaw, 03-236 Poland; 4 Department of Chemistry, University of Life Sciences, Akademicka 15, Lublin, 20-950 Poland

**Keywords:** 2-(2,4-dihydroxyphenyl)-4H-benzofuro[3,2-d][1,3]thiazin-4-one, 1,3-thiazine derivatives, Colorectal cancer, Anti-proliferative action, Cell cycle arrest

## Abstract

In this paper, we present the biological effect of the newly synthesized 2-(2,4-dihydroxyphenyl)-4*H*-benzofuro[3,2-*d*][1,3]thiazin-4-one (DPBT) on human colon adenocarcinoma cell lines (HT-29 and LS180). Additionally, DPBT cytotoxicity was examined in human colon epithelial cells (CCD 841 CoTr) and human skin fibroblasts (HSF). The studies revealed a significant decrease in the proliferation of cancer cells after exposure to DPBT at concentrations in the range of 10–100 µM. Additionally, DPBT was not toxic to normal CCD 841 CoTr and HSF cells at concentrations that induced inhibition of cancer cell proliferation. The nature of the anti-proliferative action of DPBT in the cell cycle progression in colon cancer cells and the expression of proteins involved in this process were examined by flow cytometry and western blotting, respectively. The investigations demonstrated higher sensitivity of LS180 than HT-29 to the DPBT treatment. The anti-proliferative action of DPBT in LS180 was attributed to cell cycle arrest in the G_1_ phase via up-regulation of p27^KIP1^ and down-regulation of cyclin D1 and CDK4 proteins.

## Introduction

Despite the intensive progress in modern medicine and related sciences, cancer is one of the major public health problems worldwide. Colorectal cancers (CRC) (Lv 2017) are highly proliferative and invasive human neoplasms. On a global scale, CRC is the fourth most commonly diagnosed and leading cause of cancer death in men and women (Torre et al. 2015; Siegel et al. 2016). The underlying causes of tumor progression are both genetic and epigenetic changes and certain non-genetic factors affecting the body homeostasis. Thus, progression of carcinogenesis from dysplastic tissue to the tumor and clinical symptoms may take a few to several years. During this time, accumulation of pro-cancerous events leads to disruptions of intracellular signaling pathways involved in cell viability and proliferation (Sherr and Bartek 2017; Chatterjee et al. 2017). Both surgical intervention (Mutch and Wells 2016) and chemotherapy (Rattner and Bathe 2017) are commonly and widely used in the case of CRC. Nevertheless, the increasing incidences of these diseases as well as the lack of an effective and safe treatment strategy encourage scientists to design and develop new chemotherapeutic agents (Kalyanaraman 2017).

1,3-thiazine derivatives are an interesting group of compounds with anticancer potential. These organic heterocyclic compounds contain one nitrogen and sulfur atom at the 1,3- position in the six-membered ring (Preet and Damanjit 2013). A number of studies have shown valuable biological activity of various 1,3-thiazine derivatives. Researchers reported that 1,3-thiazine derivatives inhibited growth of Gram positive bacteria like *Bacillus subtilis, B. cereus, Staphylococcus. aureus*, and *S. pyogenes*, as well as Gram negative bacteria like *Escherichia coli, Proteus vulgaris, Pseudomonas aeruginosa, P. fluorescens, P. phaseolicola, Klebsiella pneumonia*, *Mycobacterium tuberculosis*, and *M. phlei* (Koketsu et al. 2002; Sayed et al. 2006; Peng et al. 2015). Furthermore, some 1,3-thiazine derivatives exhibited antifungal action against *Candida albicans, Aspergillus niger, A. fumigates, Fusarium oxysporum, Rhizopus sp., Microsporum gypseum*, and *Penicillium citrinum* (Sayed et al. 2006; Siddiqui and Singh 2007; Ali and El Kazak 2010; Thanusu et al. 2010)). Antiviral potential of 1,3-thiazine derivatives was also reported (Koketsu et al. 2002; Yin et al. 2016). Furthermore, 1,3-thiazine derivatives are also known for their immunomodulatory, anti-inflammatory, analgesic, and anticonvulsant activity (Kalirajan et al. 2009; Jupudi et al. 2013; Bharath Rathna Kumar et al. 2014). Some 1,3-thiazine derivatives exhibit inhibitory action against specific molecular targets e.g. tyrosinase, (Ha et al. 2009) nitric oxide synthase, (Trofimova et al. 2008) galactokinase, (Odejinmi et al. 2011) or the AMPA (2-amino-3-hydroxy-5-methyl-4-isoxazolepropionic acid) receptor (Inami et al. 2015).

**Fig. 1:**
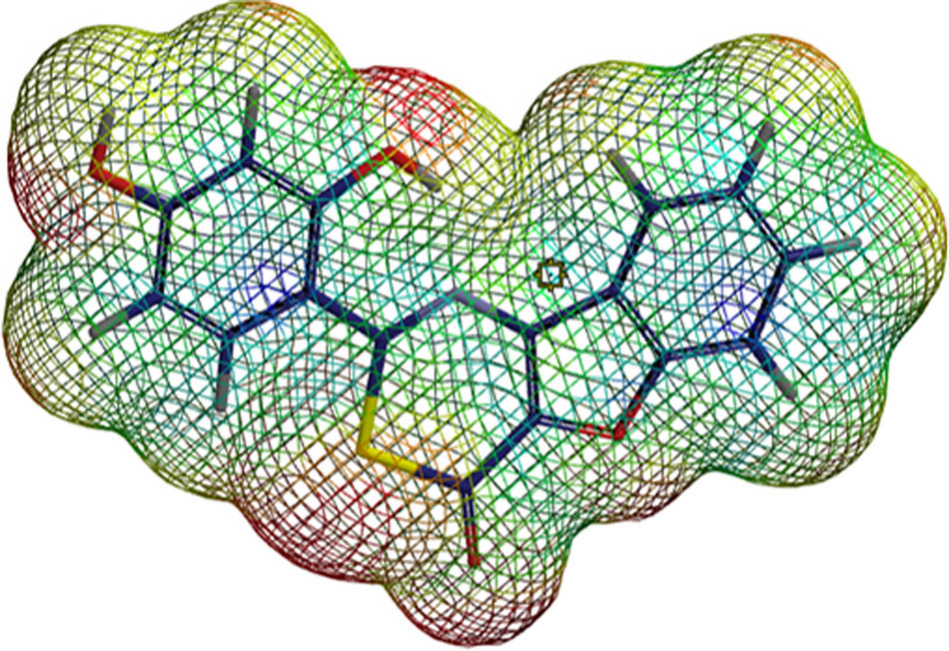
Structure of 2-(2,4-dihydroxyphenyl)-4H-benzofuro[3,2-d][1,3]thiazin-4-one (DPBT)

Given the wide spectrum of biological activity of 1,3-thiazine derivatives, the aim of this study was to determine the anticancer properties of the newly synthesized 2-(2,4-dihydroxyphenyl)-4*H*-benzofuro[3,2-*d*][1,3]thiazin-4-one (DPBT; Fig. 1) and to elucidate the molecular mechanisms of its action. The tested DPBT is characterized by a unique structure with a resorcinol substituent and a ketone group in the heterocyclic ring, which is responsible for the beneficial pharmacological properties, including the ability to interact with potential molecular targets.

## Material and methods

### Reagents

All reagents were purchased from Sigma-Aldrich (St Louis, Missouri, U.S.A) unless otherwise indicated.

### A general procedure for the synthesis of 2-(2,4-dihydroxyphenyl)-4*H*-benzofuro[3,2-*d*][1,3]thiazin-4-one (DPBT)

A mixture of 3-aminobenzofuran-2-carboxamide (1.4 mM) and sulfinylbis[(2,4-dihydroxyphenyl)methanethione] (1.4 mM) in MeOH (4 mL) was treated to reflux for 2.5–3.5 h. The hot mixture was filtered via a Buchner funnel and the solid formed during the synthesis was combined with that obtained after the filtrate concentration.

Yield: 72%, m.p.: 279–281 °C. ^1^H NMR (500 MHz, DMSO-*d*_6_) *δ*: 11.61 (s, 1H, C(2′)–OH), 10.22 (s, 1H, C(4′)–OH), 8.21 (d, *J*=9.44 Hz, 1H, C(Ar)–H), 7.81 (m, 2H, C(Ar)–H), 7.65 (m, 2H, C(Ar)–H), 7.47 (m, 1H, C(Ar)–H), 6.41 (m, 2H, C(3′,5′)–H) ppm; IR (KBr): 3443, 3334, 3167 (OH), 1652 (C=O), 1631 (C=N), 1593 (C=N), 1576 (C=C), 1233 (C–OH) cm^−1^; EI MS *m/z* (%): 371 (M^+^, 100) (for more details look at Matysiak et al. (Matysiak et al. 2015b)).

### The solutions of compound

The DPBT stock solution (100 mM) was prepared by dissolving the substance in DMSO. Afterwards, the compound was diluted in the culture medium to reach the final concentrations ranging from 10 to 100 µM. Performed studies revealed that DMSO used at the highest examined concentration was not toxic against tested cells. Final concentration of DMSO in culture medium did not exceed 0.1 %.

### Cell cultures

Experiments were carried out on human colon adenocarcinoma cell lines HT-29 and LS180, human colon epithelial cell line CCD 841 CoTr and human skin fibroblasts HSF. HT-29, LS180 and CCD 841 CoTr cell lines were obtained from the European Collection of Cell Cultures (Centre for Applied Microbiology and Research, Salisbury, UK). HSF were obtained with the outgrowth technique from skin explants from young volunteers in the Department of Virology and Immunology, UMCS, Lublin (Poland).

HT-29, LS180, CCD 841 CoTr and HSF cells were cultured in 1:1 mixture of DMEM and Nutrient mixture F-12 Ham. Media were supplemented with 10% fetal bovine serum (FBS), penicillin (100 U/mL) and streptomycin (100 mg/mL). The medium was changed every two days. The cells were grown in 25 cm^2^ flasks (Nunc, Roskilde, Denmark) and kept in a humidified atmosphere with 5% CO_2_ at 37 °C or 33 °C (CCD 841 CoTr).

### Cell proliferation assessment—MTT and BrdU assays

Cancer cells proliferation was assessed by MTT reduction and BrdU incorporation assays. The MTT assay is one of the methods for determining mitochondrial dehydrogenase activities in the living cells. In the test, the yellow tetrazolium salt (MTT 3-(4,5-dimethylthiazol-2-yl)-2,5-diphe-nyltetrazolium bromide) is metabolized by viable cells to purple formazan crystals. HT-29 and LS180 cells were plated on flat-bottom 96-well microplates at a density of 3 × 10^4^ cells/mL. The next day, the culture medium was removed and the cells were exposed to serial dilutions of DPBT at concentrations ranging from 10–100 µM. After 96 h of incubation, the cells were exposed to MTT solution (5 mg⁄mL in PBS) for 3 h. Resultant formazan crystals were solubilized overnight in SDS buffer (10% SDS in 0.01 N HCl). The color product of the reaction was quantified by measuring absorbance at a 570 nm wavelength using an Emax Miocroplate Reader (Menlo Park, CA, U.S.A). Additionally, DNA synthesis in proliferating cells was evaluated by measuring BrdU (5-bromo-2’-deoxyuridine) incorporation. Studies were performed using commercial available Cell Proliferation ELISA, BrdU (colorimetric) test (Roche Molecular Biochemicals, Mannheim, Germany). The cells (HT-29 and LS180) were plated on the 96-well microplates at a density of 3 × 10^4^ cells/mL. The following day, the culture medium was removed and the cells were exposed to serial dilutions of DPBT (10 to 100 µM). The level of DNA synthesis was quantified after 72 h by measuring BrdU incorporation according to the manufacturer’s instructions. Cell proliferation (%) for MTT and BrdU were expressed as a percentage relative to the untreated control cells (Vega-Avila and Pugsley 2011; Stepanenko and Dmitrenko 2015).

### Cell viability assessment—NR, MTT and LDH assays

In order to verify the cytotoxicity of DPBT against human colon epithelial cells CCD 841 CoTr and human skin fibroblasts (HSF) cells, neutral red (NR) and MTT cell viability assays were applied. The NR (3-amino-7-dimethylamino-2-methylphenazine hydrochloride) assay determines the accumulation of neutral red dye in the lysosomes of viable, uninjured cells. The CCD 841 CoTr and HSF cells were plated on 96-well microplates at a density of 5 × 10^5^ cells/mL. The next day cells were exposed to serial dilutions of DPBT (10–100 µM) prepared in medium with a reduced content of serum (2% FBS). After 24 h of incubation, NR and MTT cell viability assays were conducted. MTT test was performed according to the protocol described above. In case of NR assay the cells were incubated with the NR reagent for 3 h, fixed with the NR fixative solution (1% CaCl_2_ in 0.5% formalin) for 3 min at room temperature, and solubilized in 1% acetic acid in 50% ethanol under shaking for 20 min. Absorbance was measured at 550 nm using an EL800 Microplate Reader (BioTek Instruments, Winooski, Vermont, U.S.A).

In order to verify the cytotoxicity of DPBT against human colon epithelial CCD 841 CoTr, lactate dehydrogenase viability assay was applied. The test is based on measurement of lactate dehydrogenase (LDH) released into the culture medium upon damage to the plasma membrane. LDH activity is determined by several enzymatic reactions whereby the tetrazolium salt is reduced to formazan. A lactate dehydrogenase kit (In vitro Toxicology Assay Kit, Lactate Dehydrogenase Based) was used to quantify LDH release. The cells were plated on 96-well microplates at a density of 5 × 10^5^ cells/mL. The next day cells were exposed to serial dilutions of DPBT (10–100 µM) prepared in medium with a reduced content of serum (2% FBS). The culture supernatants were collected in new 96-well microplates, which were used to perform the LDH assay according to the manufacturer’s instructions. The data were expressed as a percentage relative to the untreated control cells of three independent NR, MTT and LDH experiments for each assay (Fotakis and Timbrell 2006; Repetto et al. 2008).

### Analysis of cell cycle by flow cytometry—PI staining

Propidium iodide (PI) staining is a method for analysis of cellular DNA content by flow cytometry. The HT-29 and LS180 cells were seeded on 6-well microplates at a density of 5 × 10^5^ cells/mL. Next day the cells were exposed to 25; 50; 75 µM of the DPBT for the next 24 h (at 37 °C and 5% CO_2_). Then the medium was removed and the cells were washed with PBS and collected by a 5 mM EDTA/PBS solution. After centrifugation (10 min; 500*g*), the cells were fixed with 70% ice-cold ethanol and then stored at −20 °C. PI/RNase (BD Life Sciences, Franklin Lakes, New Jersey, U.S.A) staining was performed directly before flow cytometric analysis according to the manufacturer’s instructions. Cell cycle assessment was performed using BD FACSCalibur. The PI fluorescence intensity of individual nuclei was determined and at least 10000 events were measured within an acquisition rate 100–300 events/s. The cell cycle analyses were performed with the use of software CellQuest Pro Version 6.0. for the Macintosh operating system (Pozarowski and Darzynkiewicz 2004).

### Western blotting analysis

The HT-29 and LS180 cells were plated on flat-bottom 6-well dishes in 2 mL of medium and cultured overnight. The following day, the cultures were exposed to serial dilutions of DPBT (25; 50; 75 µM) for 24 and 48 h. Next, cells were washed with ice-cold PBS, harvested (5 mM EDTA in PBS), and lysed for 1 h in ice-cold lysis buffer consisting of 1% NP40, 0.5% sodium deoxycholate, 0.1% SDS, 1 mM EDTA, 1 mM EGTA, 1 mM Na_3_VO_4_, 20 mM NaF, 0.5 mM DTT, 1 mM PMSF, and protease inhibitor cocktail in PBS, pH 7.4. After centrifugation in 4 °C (14,000*g* for 10 min) the protein content in the supernatants was determined with the BCA Protein Assay Kit (Pierce Biotechnology, Waltham, Massachusetts, U.S.A). Next, supernatants were collected and solubilized in 6× Laemmli sample buffer (0.5 M Tris-HCl, pH 6.8, 30% glycerol, 10% SDS, 5% β-mercaptoethanol, 0.012% bromophenol blue). Samples were boiled for 5 min, and stored at −20 °C. Gel electrophoresis of proteins were performed on 10–12% polyacrylamide gels (SDS-PAGE) transferred to PVDF (Millipore, Billerica, Massachusetts, U.S.A) membranes and blocked (1 h at room temperature) with 10% nonfat dry milk in TBS-0.1%/Tween 20 (TBST). To detect the protein level, the PVDF membrane was probed overnight at 4 °C with the following antibodies: cyclin D1, cyclin A2, cyclin B1, p27^KIP1^, CDK4 and β-actin (Cell Signaling Technology, Beverly, Massachusetts, U.S.A) (1:1000 in TBST). Then the membranes were washed in TBST buffer and incubated with secondary antibody conjugated with horseradish peroxidasefor (1:1000, Cell Signaling Technology) for 1 h at room temperature. The visualization of the proteins was performed using an enhanced chemiluminescence detection system (Pierce, Rockford, IL, U.S.A). The amount of protein was densitometrically determined using ImageJ (developed by Wayne Rasband) software (Kurien and Scofield 2006).

### Statistical analysis

Statistical analyses were performed using GraphPad Prism 5.0 (GraphPad Software Inc., California, U.S.A) plotted as the mean ± SD. ANOVA with Tukey post hoc test and column statistics were used for comparisons (*, *p* < 0.05; **, *p* < 0.01; ***, *p* < 0.001). IC_50_ (concentration causing proliferation inhibition by 50% compared to the control) was calculated from the nonlinear regression (log(inhibitor) vs. normalized response-variable slope) using GraphPad Prism 5.0.

## Results

### DPBT inhibits HT-29 and LS180 cancer cells proliferation

**Fig. 2:**
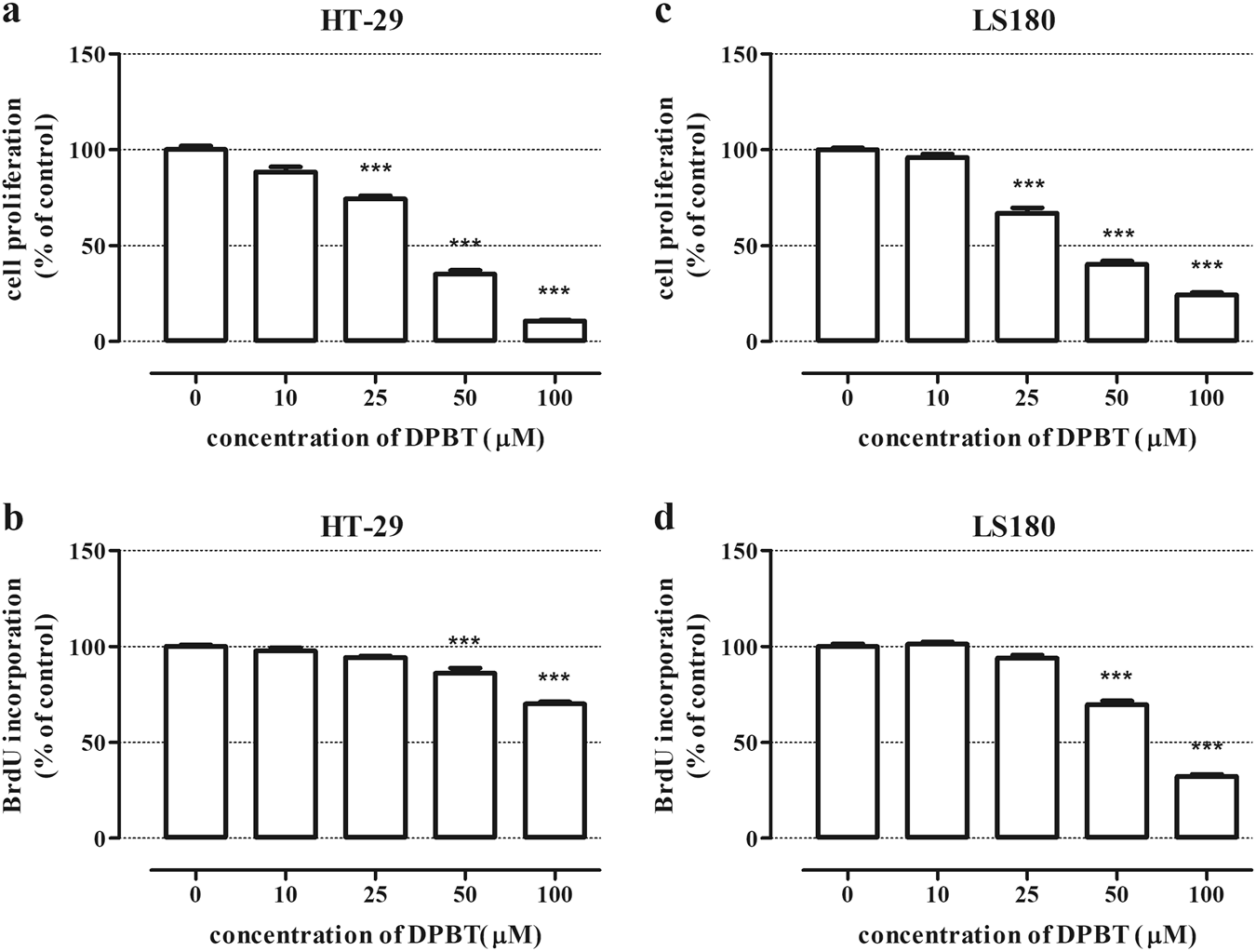
Anti-proliferative effect of DPBT. Human colon adenocarcinoma cells (HT-29 and LS180) were exposed to the tested compound at concentrations ranging from 0 to 100 µM for 96 h (**a**, **c**—MTT assay) or 72 h (**b**, **d**—BrdU assay). Cell proliferation was measured photometrically by means of the MTT assay (**a**, **c**) or BrdU colorimetric immunoassay (**b**, **d**). The proliferation of untreated control cells was calculated as 100%. The results represent the mean ± SD and were analyzed with one-way ANOVA test and Tukey’s Multiple Comparison Post-test (***p < 0.001 was considered statistically significant)

In our study, the anti-proliferative effect of a 1,3-thiazine derivative DPBT was determined using an MTT BrdU assays, which were performed on the human colorectal adenocarcinoma (HT-29 and LS180) cell lines. After 96 h of exposure, the tested compound exerted a dose-dependent anti-proliferative effect on each cancer cell line in the MTT test. The IC_50_ value of DPBT for the HT-29 and LS180 cell lines was estimated at 40.81, 29.60 µM, respectively. Significant inhibition of cancer cell proliferation was initially observed after the use of DPBT at a concentration of 25 μM in both HT-29 and LS180 cells (Fig. 2a, c). As assessed by the MTT assay, the cell division rate clearly diminished along with the increasing concentrations of DPBT. Thus, we decided to confirm the anti-proliferative activity of DPBT in the more sensitive and specific BrdU test (Fig. 2b, d). As expected, 72 h of treatment with DPBT reduced DNA synthesis in all the investigated cell lines. In the assays, the IC_50_ value of DPBT for the HT-29 and LS180 cell lines was estimated at 152.2 and 65.43, respectively.

### DPBT exhibits no toxicity to normal HSF and CCD 841 CoTr cells

**Fig. 3:**
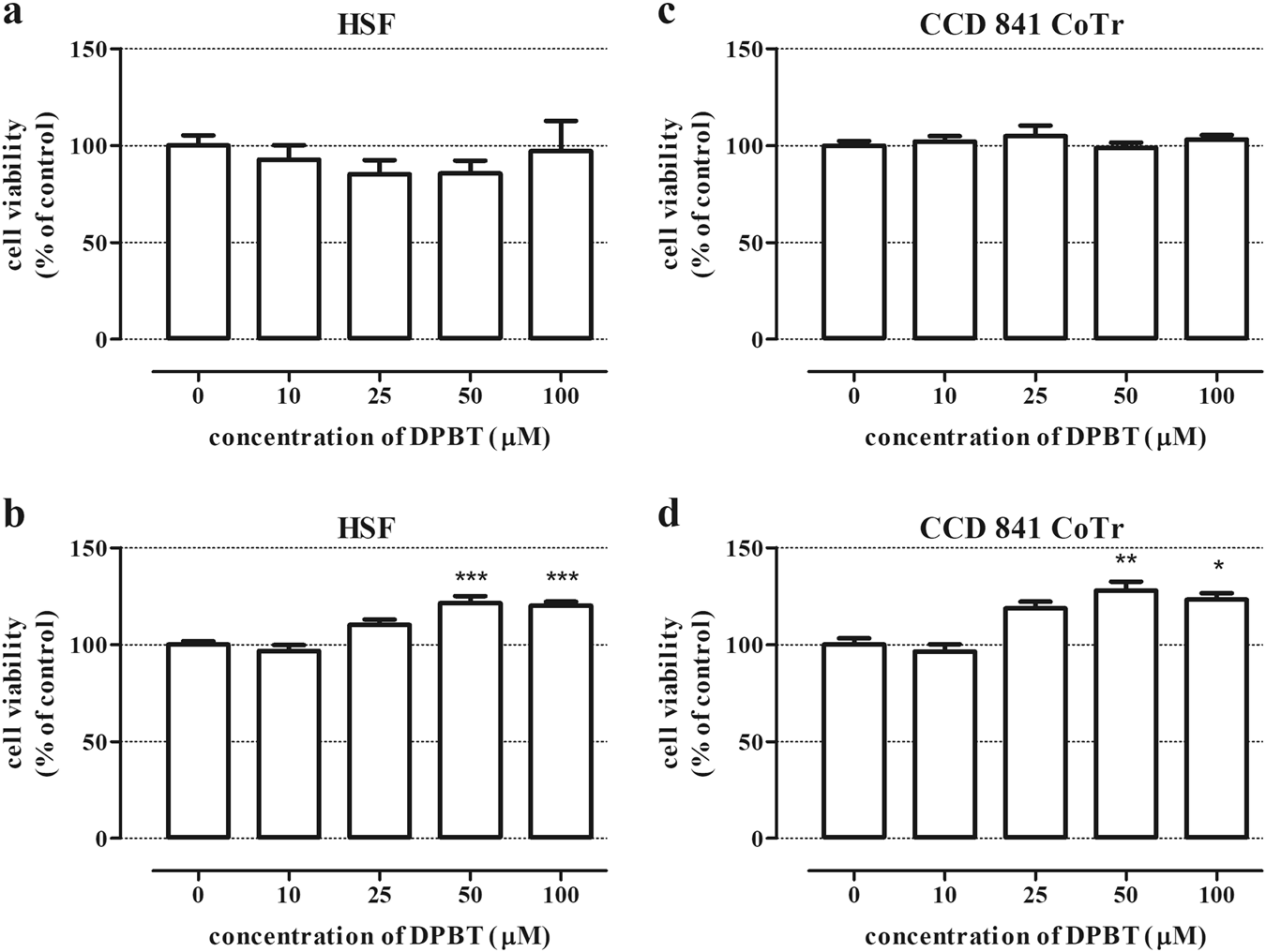
DPBT influence on the viability of human fibroblasts (HSF) and human colon epithelial cells (CCD 841 CoTr). The cells were treated with DPBT at concentrations ranging from 0 to 100 µM for 24 h. The cell viability was measured photometrically by means of the NR assay (**a**, **c**) or the MTT assay (**b**, **d**). The cell viability of untreated control cells (0) was calculated as 100%. The results represent the mean ± SD and were analyzed with one-way ANOVA test and Tukey’s Multiple Comparison Post-test (*p < 0.05, **p < 0.01, and ***p < 0.001 were considered statistically significant)

**Fig. 4:**
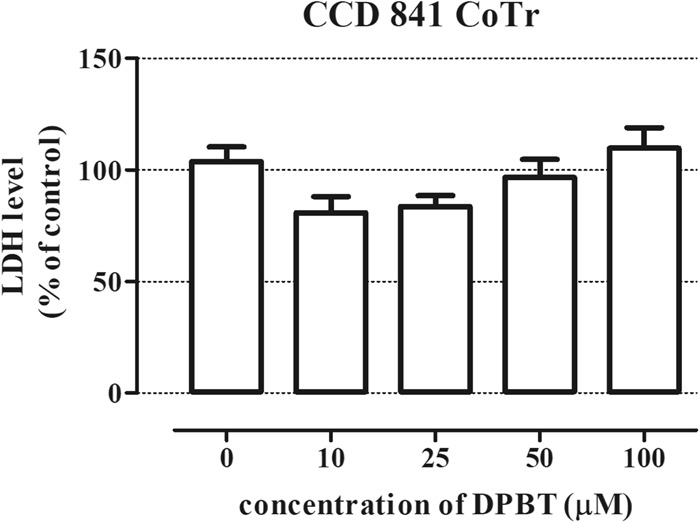
Cytotoxic effect of DPBT in human colon epithelial cells (CCD 841 CoTr). The cells were treated with DPBT at concentrations ranging from 0 to 100 µM for 24 h. Cell membrane integrity was measured photometrically by means of the LDH assay. The results represent the mean ± SD and were analyzed with one-way ANOVA test and Tukey’s Multiple Comparison Post-test (*p < 0.05, **p < 0.01, and ***p < 0.001 were considered statistically significant)

In our study, the toxic effect of DPBT was determined using the NR and MTT assays, which were performed on the human skin fibroblasts HSF and human colon epithelial cell line CCD 841 CoTr. The NR test showed that the viability of the normal HSF and CCD 841 CoTr cells was unaffected in the entire concentration range (10–100 µM) of DPBT (Fig. 3a, c). Additionally, the cytotoxicity of DPBT was evaluated using the LDH assay on CCD 841 CoTr cells. The LDH assay data confirmed the results obtained in the NR assay in the case of CCD 841 CoTr cells (Fig. 4). In contrast, treatment of the HSF and CCD 841 CoTr cells with concentrations ranging from 50 to 100 µM caused a slight but statistically significant increase in mitochondrial dehydrogenase activities in the MTT assay (for 24 h of incubation), (Fig. 3b, d).

### DPBT disturbs LS180 cell cycle progression

**Fig. 5:**
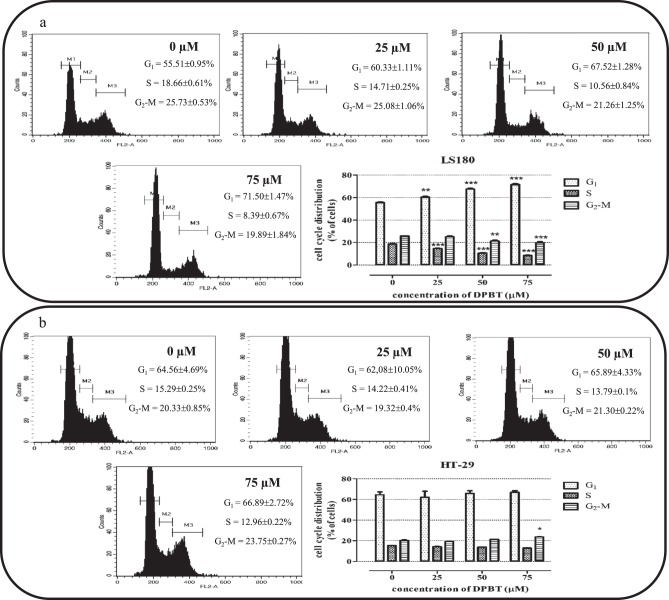
Effect of DPBT on cell cycle progression. After 24 h of LS180 **a** and HT-29 **b** cell treatment with the increasing concentrations of DPBT (25, 50, 75 µM), the cells were stained with propidium iodide and analyzed with flow cytometry. Representative cell cycle histograms demonstrating the DNA content and the percentage of cells in the G_1_ (gate M1), S (gate M2), and G_2_-M (gate M3) phases are shown. Untreated control cells (0). The percentage of cells in the G_1_, S, G_2_-M phases was expressed as mean ± SD and was analyzed with one-way ANOVA test and Tukey’s multiple comparison post-test (*p < 0.05, **p < 0.01, ***p < 0.001 were considered statistically significant)

The reduction of cancer cell proliferation is often associated with cell cycle inhibition. Thus, the ability of DPBT to modulate the cell cycle progression was examined by flow cytometry using the PI staining assay. The studies indicated prominent changes in the LS180 cell cycle progression. The increasing concentrations of DPBT influenced the cell cycle progression in a dose-dependent manner, showing statistically significant cell accumulation in the G_1_ phase and a decrease in the cell number in the S and G_2_-M phases (Fig. 5a). After the 24 h exposure, the highest concentration of DPBT (75 μM) increased the percentage of cells in the G_1_ phase by 15.99% vs. control cells. Additionally, the percentage of cells in S and G_2_-M was decreased by 10.27 and 5.84%, respectively. In contrast, the HT-29 cells were less sensitive and no significant changes were observed in the G_1_ and S phases of cell cycle progression after exposure to DPBT. Only 75 μM DPBT increased the number of HT-29 cells in G_2_-M by 3.42% vs. the control (Fig. 5b).

### Effect of DPBT on the levels of cell cycle regulators

**Fig. 6:**
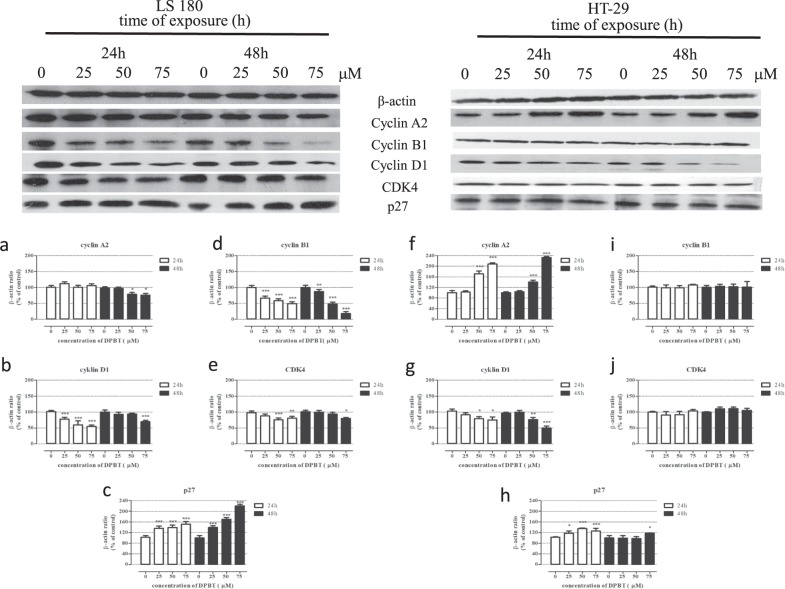
Influence of DPBT on the cell cycle regulatory proteins in human colon adenocarcinoma cell lines LS180 and HT-29. The cells were incubated with the indicated concentrations of DPBT (25, 50, 75 µM) for 24 or 48 h and analyzed by western blotting using target specific antibodies. The equivalent amount of protein was verified by reprobing the blot with anti-β-actin antibody (internal control). Representative western blots (upper panel) with densitometric analyses (lower panel). Representative blots of whole LS180 (**a**–**c**) and HT-29 (**f**–**h**) cell lysates. Quantitative analysis of band intensities by means of densitometric analysis: cyclin A2 (**a**, **f**), cyclin B1 (**d**, **i**), cyclin D1 (**b**, **g**), CDK4 (**e**, **j**) and p27^KIP1^ (**c**, **h**). The results of the densitometric analysis are presented as mean ± SD and were analyzed with one-way ANOVA test and Tukey’s multiple comparison post-test (*p < 0.05, **p < 0.01, and ***p < 0.001 were considered statistically significant)

To examine the impact of DPBT on the expression of proteins regulating the cell cycle, the western blotting method was used. The analysis of proteins in the LS180 cell line under the DPBT exposure showed a dose-dependent increase in p27^KIP1^ expression and a decrease in CDK4 and cyclin D1 expression after 24 and 48 h of treatment. In our study, the 24 h exposure of the LS180 cells to 75 μM of DPBT resulted in almost 50% reduction of the cyclin D1 level (Fig. 6b), 20% reduction of the CDK4 level (Fig. 6e), and a 50% increase of the p27^KIP1^ level (Fig. 6c), in comparison to the untreated cells. Furthermore, 75 μM DPBT resulted in reduction of the expression of cyclin B (Fig. 6d) and cyclin A2 (Fig. 6a) by 51% (24 h treatment) and 25% (48 h treatment), respectively.

The analysis of proteins in the HT-29 cell line under the DPBT exposure showed increase in cyclin A2 and p27^KIP1^ expression and decrease in cyclin D1 expression after 24 and 48 h of the treatment. After 24 h of cell exposure to 75 μM DPBT, there was an increase in the expression of cyclin A2 (108% vs control) (Fig. 6f), p27^KIP1^ (25% vs control) (Fig. 6h), and approximately 25% reduction of the cyclin D1 level (Fig. 6g). In contrast to the LS180 cells, no statistically significant changes in the expression of cyclin B1 (Fig. 6i) and CDK4 (Fig. 6j) were observed.

## Discussion

Malignant neoplasms develop and disturb homeostasis through uncontrolled cell proliferation. Thus, inhibition of fast dividing cancer cells is the basic feature of chemotherapeutic agents. Therefore, it is essential to progress the research of novel anticancer agents to treat malignant diseases. So far, DPBT has been studied relatively weakly in the area of anticancer activity. Our results of the MTT and BrdU experiments revealed that DPBT had significant anti-proliferative activity in the case of the HT-29 and LS180 colon cancer cell lines. Recently, we have revealed high activity of DPBT against bladder cancer cells HCV29T (the IC_50_ value was 20.37 µM for sulforhodamine B assay) and some anti-proliferative features of benzofuro-1,3-thiazinone resorcinol hybrids against the A549 (human non-small cell lung carcinoma), T47D (human breast cancer), and SW707 (human rectal adenocarcinoma) cell lines (Matysiak et al. 2015b). We also detected comparable anticancer activity of some thieno-1,3-thiazin-4-one derivatives against lung cancer A549, colon adenocarcinoma HT-29, and glioma C6 cells (Matysiak et al. 2015a). As can be seen, 1,3-thiazine derivatives exhibit significant anti-proliferative potency in a broad range of cancer cells.

Anticancer drugs are very often toxic to normal highly proliferative tissues such as bone marrow, mucosa as well as musculoskeletal and nervous system (Lee et al. 2014; Wen and Li 2016; Banach et al. 2017). Thus, examination of the cytotoxic effect of new potential anticancer agents is a key step in anticancer drug screening. Our initial studies showed that DPBT was non-toxic to normal colon epithelial cells CCD 841 CoTr in the entire concentration range. Additionally, treatment of the HSF and CCD 841 CoTr cells caused statistically significant increase in mitochondrial dehydrogenase activities in the MTT assay (for 24 h of incubation), which may suggest protective or trophic properties of DPBT. Reduction of MTT is an established method for evaluating the viable cell ratio in proliferation and toxicity studies. However, it is known that substances that alter the mitochondrial metabolism of cells may affect the rate of MTT reduction into formazan and, consequently, have an impact on the MTT assay read out (Riss et al. 2013; Stepanenko and Dmitrenko 2015). Thus, the observed phenomenon requires further studies in order to prove the beneficial effects of DPBT against normal cells.

Further analysis was focused on the DPBT influence on cancer cell cycle and proteins responsible for regulation of cell cycle progression in order to describe fully the molecular mechanism of the anticancer action of the tested compounds. It is known that the eukaryotic cell cycle is tightly regulated. Phase G_1_ is controlled by assembly and activation of complexes of cyclins (mainly D and E) and relevant serine–threonine protein kinases like cyclin-dependent kinases CDK4 and CDK6. These complexes lead to phosphorylation (and then inhibition) of the tumor suppressor protein Rb and, consequently, lead to release of E2F-family transcription factors. E2Fs are involved in the proliferation process through control of the expression of a series of genes essential for the progression of the S and G_2_-M phases, including cyclin A (a key regulator of DNA replication) and cyclin B (a key regulator of cell entry into mitosis) genes. The activity of cyclin/CDK complexes is negatively regulated by families of low molecular CDK inhibitors like the p27^KIP1^ protein. A diminished proliferation ratio as a consequence of cell cycle arrest is often related to down-regulation of cyclins and cyclin-dependent kinases with accompanying over-expression of CDK inhibitors (Viallard et al. 2001; Cheng 2004; Hnit et al. 2015).

The studies revealed that the altered cell distribution in the cell cycle after the DPBT exposure was significantly more pronounced in the LS180 cells than in the HT-29 cells. We have demonstrated that the tested DPBT caused dose-dependent cell cycle arrest in the G_1_ phase. Consequently, the molecular mechanism of the anticancer properties of DPBT was further examined in LS180 cells. The down-regulation of cyclin D1/CDK4 and up-regulation of p27^KIP1^ proteins by DEBT might be responsible for the decrease in the expression of S phase-related E2F transcription factors, which explains the reduction of cyclin B1 and A2 protein level observed in the LS180 cells. Recently, we have described a similar anti-proliferative mechanism of the anticancer action of BChTT (6-tert-butyl-2-(5-chloro-2,4-Dihydroxyphenyl)-4*H*-thieno[3,2-*d*][1,3]thiazin-4-one), which, via activation of p38 kinase, decreased cyclin D1 expression and, consequently, induced cell cycle arrest in the G_1_ phase. Furthermore, BChTT was not toxic to normal cells, including skin fibroblasts, hepatocytes, and oligodendrocytes (Juszczak et al. 2016).

The studies performed on the HT-29 cells revealed a different model of DPBT action. Consistent with these observations, it is possible that the expression of cyclin A2 in the late G_1_ phase accelerated cell entry into the S-phase (cyclin A2/Cdk2 complexes are responsible for activation of DNA polymerase α and, consequently, DNA replication). At the same time, DPBT inhibited G_1_-S transition by an increase in p27^KIP1^ and a decrease in the cyclin D1 level. Thus, these opposite effects may be responsible for the lower sensitivity of the HT-29 cells to DPBT. Additionally, the slight accumulation of the HT-29 cells treated with DPBT in the G_2_ phase may be correlated with the up-regulation of the p27^KIP1^ level by the tested compounds. It has been shown that in some cases p27^KIP1^ might also function as an inhibitor of G_2_-M transition by down-regulation of CDK1 (Sharma and Pledger 2016). Thus, DPBT may inhibit HT-29 cell proliferation by up-regulation of p27^KIP1^.

## Conclusions

To sum up, DPBT acts in a specific way affecting rapidly proliferating CRC cells. However, the investigation results indicated higher anticancer potential of DPBT against LS180 colon cancer cells. Our results suggest that up-regulation of the cell cycle G_1_ phase inhibitor p27^KIP1^ and down-regulation of cyclin D1 and CDK4 may be critical factors for the anti-proliferative action of DPBT in the LS180 cell line. Thus, the DPBT should be further studied as a chemotherapeutic agent for some types of CRC. Further pre-clinical studies are needed to confirm the safety and efficacy of the compound.
